# The Characteristics, Prognosis, and Risk Factors of Lymph Node Metastasis in Early Gastric Cancer

**DOI:** 10.1155/2018/6945743

**Published:** 2018-05-02

**Authors:** Xuan Li, Shiyu Liu, Jin Yan, Lei Peng, Meihong Chen, Jiajia Yang, Guoxin Zhang

**Affiliations:** ^1^Department of Gastroenterology, The First Affiliated Hospital of Nanjing Medical University, Nanjing 210000, China; ^2^Department of Gastroenterology, The First School of Clinical Medicine of Nanjing Medical University, Nanjing 210000, China; ^3^Department of Gastroenterology, First People's Hospital of Xuzhou, Xuzhou 221007, China

## Abstract

**Background:**

Lymph node metastasis (LNM) is the most important risk factor for endoscopic treatment in early gastric cancer (EGC) patients. We aimed to investigate the rate of LNM, the risk factors, and the prognosis of EGC patients with LMN.

**Methods:**

A total of 10,039 patients who underwent gastrectomy with lymphadenectomy were reviewed between January 2010 and December 2015 at Jiangsu Province Hospital in China. Among them, we identified 1004 (10%) EGCs. First, endoscopic and clinicopathological features related to LNM were analyzed, and then risk factors for LNM were identified using univariate and multivariate analysis. Finally, the short- and long-term outcomes were compared between the groups.

**Results:**

LNM occurred in 123 (12.3%) EGCs. Most of EGCs were male (*n* = 720, 71.7%) and mean age was 59.65 ± 11.09 years. The rate of *H. pylori* infection was 78.0% (783/1004). LNM was significantly associated with age, sex, location, lesion size, macroscopic type, depth of invasion, differentiation type, histological morphology, lymphovascular invasion (LVI), and TMN stage. By multivariate analysis, significant independent risk factors for LNM in EGC were identified as following: male sex (OR 2.365, *P* = 0.035), age ≦ 40 (OR 0.055, *P* = 0.012), depressed type (OR 2.721, *P* = 0.013), submucosa invasion (OR 2.987, *P* = 0.032), LVI (OR 5.186, *P* = 0.003), tumor located in corpora (OR 8.904, *P* = 0.047), and in angle (OR 12.998, *P* = 0.024). 86.5% were successfully followed up for 3 years. The overall 1- and 3-year survival rates in LNM group were 100% and 91.1%, respectively, and those with no LNM were 100% and 100%, respectively.

**Conclusion:**

EGCs were investigated in 10.0% of gastric cancer, which LNM occurred in 12.3% of EGC. Independent risk factors of LNM included male sex, age (>40), the depth of invasion, LVI, and tumor located in corpora or angle. The 3-year overall survival rate was greater in EGC patients without LNM.

## 1. Introduction

Gastric cancer (GC) is one of the most prevalent malignancies in Eastern Asia [1], and the incidence of GC has been increasing in keeping with the aging [2]. In China, about three million new cases were investigated and two million patients with GC died per year [3]. As we have known, GC is classified into advanced gastric cancer (AGC) and early gastric cancer (EGC). EGC is defined as tumor confined to the mucosa and submucosa of the stomach, with or without regional lymph node metastases [4]. The mortality of GC is high associated with the stage of GC; thus, the early diagnosis and treatment of GC is the most effective way to decrease the mortality.

With the development of gastroscopy, the incidence of EGC has been gradually increased, and endoscopic surgical techniques, including endoscopic mucosal resection (EMR) and endoscopic submucosal dissection (ESD), have been regarded as an alternate treatment to traditional surgical techniques for EGC. Quality of life can be improved due to less complications associated with surgical gastrectomy. According to the standard criteria of the Japanese Gastric Cancer Association (JGCA) guidelines [5, 6], we can resect tumors by the use of ESD, which satisfied the following criteria: (1) confined to the mucosa, (2) less than 2 cm in diameter, (3) without intramural ulcers, and (4) histologically differentiated. However, several studies showed that the rate of lymph node metastases (LNM) was up to 25.4% [7, 8], due to no treatment of lymph nodes in ESD. For the recurrence rate, LNM has been proved to be one of the most important prognostic factors [9]. Therefore, detection risk factors of EGC with LNM is essential to improve the prognosis of EGC patients with LNM.

In the present study, we aimed to analyze the clinical characters of EGCs, investigate the rate of LNM, and clarify risk factors of EGC with LNM.

## 2. Methods

### 2.1. Patients

**Figure 1:**
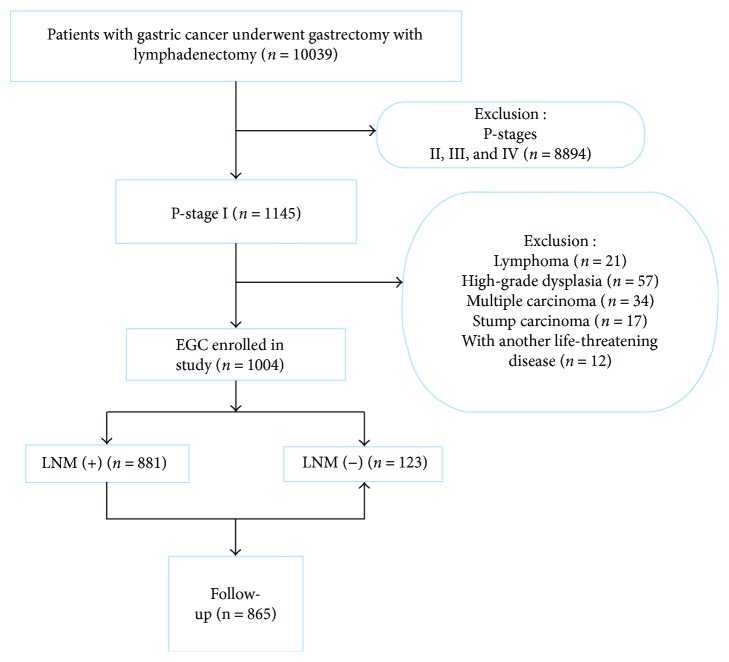
Flow diagram showing the study design and population. EGC: early gastric cancer; LNM: lymph node metastasis.

We retrospectively reviewed all patients who diagnosed with gastric cancer and underwent gastrectomy with lymphadenectomy between January 2009 and December 2014 at Jiangsu Province Hospital in China. A total of 10,039 patients were reviewed. Two experienced pathologists individually examined the histological slides of all EGCs. All included patients receive conventional white light upper endoscopic examination with biopsy before gastrectomy. Patients were excluded if they satisfied the following excluded criteria: (i) advanced-stage gastric cancer (*n* = 8894), (ii) lymphoma (*n* = 21), (iii) high-grade dysplasia (*n* = 57), (iv) multiple carcinoma (*n* = 34), (v) stump carcinoma (*n* = 17), and (vi) with another life-threatening disease (*n* = 12). Finally, a total of 1004 EGCs were included in this study (Figure 1).

This study protocol was approved by the Medical Ethics Committee of The First Affiliated Hospital of Nanjing Medical University.

### 2.2. Endoscopic Procedure and Data Collection

We further collected the clinical data, endoscopic features, and pathological data of all included patients and followed up them. The clinical data included age, sex, *Helicobacter pylori* (*H. pylori*) infection, smoking, and drinking. As for *H. pylori* infection, if any one of the following tests was positive, rapid urease test, histologic examination from forceps biopsy specimen, or serologic test, the patient was considered infected. Then, two experienced endoscopists individually reviewed endoscopic images and reports. Macroscopic types, lesion size, and location were obtained from the endoscopic examination. The macroscopic features of tumors were divided into three types: I, II, and III. The II type was further divided into elevated with a rough surface (IIa), flat (IIb), and flat with an eroded surface (IIc) [10, 11]. The common ground between IIc and III is depressed. The location of lesions was separated into upper part of stomach (the cardia and the upper half of the stomach body) and lower part of stomach (the antrum and the lower half of the stomach body). Tumors were also described as small, middle, and large tumor according to the diameters of 20 mm and 30 mm.

### 2.3. Pathological Examination

In the pathological examinations, histologic types, depth of invasion, lymphovascular invasion (LVI), LNM, and perineural invasion were extracted. As for histological morphology, patients were categorized into the following types based on the World Health Organization (WHO) criteria: adenocarcinoma, mucinous carcinomas, signet ring cell, carcinoma with lymphoid stroma, neuroendocrine carcinoma, adenosquamous carcinoma, and so on [12]. Histologic types were divided into differentiated type and undifferentiated type based on the criteria of the Japanese Gastric Cancer Association (JGCA) [10]. We also classified into well-differentiated, moderately differentiated, poorly differentiated adenocarcinoma. The stage of disease was classified on the basis of topography, lymph node, and metastasis.

### 2.4. Statistical Analysis

All statistical analyses were conducted with SPSS version 21.0 (SPSS Inc., Chicago, IL, USA). The mean ± standard deviation and number with percentage were used for the continuous and categorical variables, respectively. For continuous variables, group comparisons were calculated by Student's *t*-test. For categorical variables, group comparisons were performed with Pearson's chi-square test or Fisher's exact test. Logistic regression analysis was used to evaluate the risk of lymph node metastasis. The 1- and 3-year overall survival rates were demonstrated by the log-rank test. *P* values less than 0.05 were considered statistical significance.

## 3. Results

### 3.1. Clinical Characteristics

Firstly, a total of 10,039 patients with GC were reviewed. After further assessment, a total of 1004 (10.0%) patients with EGC were enrolled in our study, including LNM (*n* = 123) and without LNM (*n* = 881). Finally, the rate of EGC detection was 10.0%, and the LNM rate was 12.3%. Data on clinical characteristics for EGCs were extracted in Table 1. The mean age was 59.65 ± 11.09 years. EGC was predominantly found in gerontism. Most of the patients were male (*n* = 720, 71.7%). The rate of *H. pylori* infection was 78.0% (783/1004).

### 3.2. Comparison of Endoscopic Features

Endoscopic features of included patients are presented in Table 2. The smallest size of tumor was 1 mm, while the largest was 90 mm. The mean lesion size was 19.4 mm ± 11.6. As for location, we found the largest number of EGC was in the antrum (*n* = 446), followed by the cardia (*n* = 217), corpora (*n* = 207), angle (*n* = 124), and fundus (*n* = 10). Overall, most of the lesions were found in the lower part of the stomach (76.1%, 764/1004). With regard to macroscopic features, EGC occurred more frequently on the type of depressed (66.7%, *n* = 670), followed by flat (24.0%, *n* = 241) and elevated (9.3%, *n* = 93). Furthermore, IIc (57.5%, *n* = 382) predominantly occurred in the type of depressed.

### 3.3. Pathological Features

The pathological features of the included patients are listed in Table 3. Patients with the following types in our study: adenocarcinoma (*n* = 793), mucinous carcinomas (48), signet ring cell (*n* = 155), neuroendocrine carcinoma (*n* = 5), and carcinoma with lymphoid stroma (*n* = 3). Most histologic types of EGC were differentiated type (72.1%, *n* = 724) based on the JGCA criteria. According to WTO criteria, the differentiation of EGC mostly was well-to-moderately differentiated carcinoma (82.1%, *n* = 824), while 180 EGCs were poorly differentiated carcinoma. The number of mucosa invasion in EGCs was analogous to that of submucosa invasion (*n* = 504/501); however, submucosa invasion was observed in 76.4% (*n* = 94) patients with LNM. LVI was identified in 60 patients, in which 30 (3.4%) had LNM and 30 (24.3%) were without LNM. Only 7 patients occurred perineural invasion, which was observed more often in patients without LNM (*n* = 6). As for TMN staging, all of patients without LNM were stage 0, while all patients who were stage I had LNM.

### 3.4. Multivariate Analysis of LNM

By multivariate analysis, significant independent risk factors for LNM in EGC were identified as following: male sex (OR 2.365, 95% CI 1.064–5.257, *P* = 0.035), age ≦ 40 (OR 0.055, 95% CI 0.006–0.533, *P* = 0.012), depressed type (OR 2.721, 95% CI 1.232–6.008, *P* = 0.013), submucosa invasion (OR 2.987, 95% CI 1.100–8.111, *P* = 0.032), LVI (OR 5.186, 95% CI 1.751–15.366, *P* = 0.003), tumor located in corpora (OR 8.904, 95% CI 1.029–77.07, *P* = 0.047), and tumor located in angle (OR 12.998, 95% CI 1.400–120.677, *P* = 0.024) (Table 4).

### 3.5. Long-Term Outcomes

**Figure 2:**
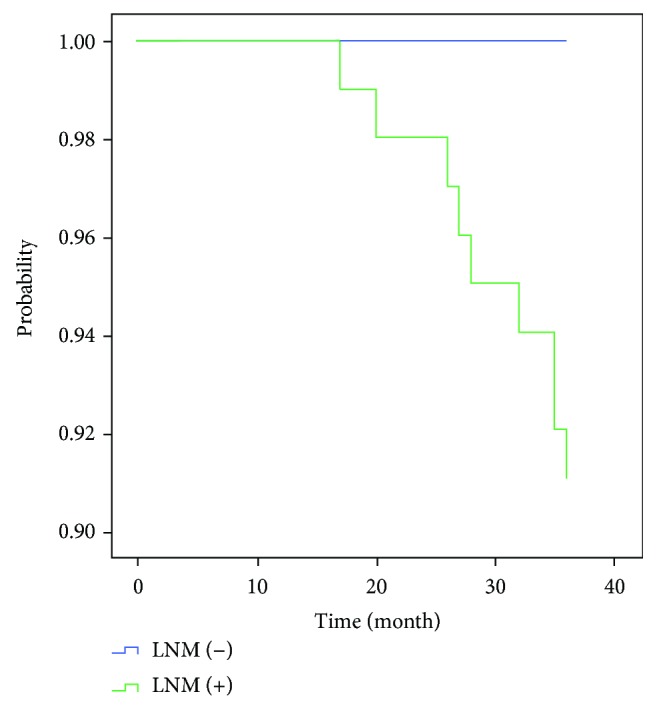
Three-year overall survival between the two groups.

Of the remaining 1004 patients, 868 (86.5%) were successfully followed up for 3 years, including 766 with LNM and 102 without LNM. In 29 (3.3%) cases of tumor recurrence, 21 (2.4%) had no LNM and 8 (7.8%) had LNM. 22 (2.5%) deaths were reported after 36 months which 7 (6.9%) had LNM and 15 (1.7%) had no LNM. The overall 1- and 3-year survival rates in LNM group were 100% and 91.1%, respectively, and those with no LNM were 100% and 100%, respectively (Figure 2). The overall 3-year overall survival rate was greater in patients without LNM (*P* < 0.001).

## 4. Discussion

Several studies in Europe reported the detection rate EGC found during GC resections was 11–15% [13, 14]; our study showed that the rate of EGC detection was 10.0%, but lower than that in Japan and South Korea (over 50%) [15]. LNM found in EGC was 12.3% in our study, in contrast with 2.5–8.6% in Japan [5, 16]. This may be due to the fact that Japan's early cancer screening policy and high-grade intraepithelial neoplasia were defined as EGC in the diagnostic criteria of Japanese standards.

In our study, to identify the clinical impact of LNM in EGC, we also compared the endoscopic and clinicopathological features between the LNM group and no LNM group. The present study showed that patients with LNM were closely associated with elderly patients, male sex, depressed type, LVI, depth of invasion, and tumor located in corpora and angle. In terms of long-term outcomes, the patients without LNM had better 3-year overall survival compared with those with LNM.

Both ESD and gastrectomy were the main methods for the treatment of EGC, which have similar efficacy in the treatment of EGC. It was suggested that part of patients underwent gastrectomy may accept unnecessary enlargement of operation, while part of patients with LNM who received ESD may not undergo lymph node dissection. Our result demonstrated that male sex was a risk factor for LNM in EGC, which was 2.365 times higher than in women. Moreover, Tian et al. also found that sex was in the prognosis of LVI in SM1 gastric cancer [17]. On the one hand, it may be related to more smoking and alcohol in male. Buckland et al. reported smoking and alcohol played an important role in development of GC [18]. On the other hand, androgen receptor is involved in many signaling pathways associated with gastric carcinogenesis [19]. As attested in some series, estrogen was connected with the proliferation and invasion of gastric cancer, which could be prevented by tamoxifen, an estrogen receptor antagonist [20, 21]. In addition, we found the incidence of gastric cancer increased with age, and by multivariate analysis, age ≦ 40 was a protective factor of LNM in EGC. It is a long process that carcinogens cause cell damage and tumor formation.

Our study also showed that depressed EGCs were primarily found with significant increased LNM risk. And we found that IIc, a combination of flat and depress type, was identified in 381 patients, which was also associated with an increased risk of LNM in many Japanese's studies [22]. In our study, the incidence of EGC was most frequent in the antrum. However, by multivariate analysis, lesions located in corpora and angle were the risk factors. Several studies also reported the mid-to-upper part of the stomach was one of the risk factors [23]. As we have known, the thickness of submucosa is thinner in the corpora and angle than in the antrum [24], and small lymphatic capillaries are directly present above the lamina mucosa [25]. Thus, an EGC lesion may be more vulnerable to occurring LNM in the corpora and angle.

The rate of lymph node metastasis was predominantly related to the LVI and the depth of invasion [26], and since lymphatic vessels were richer in deeper third of lamina propria and submucosal, it might be the reason that LVI and the depth of invasion were related to the rate of lymph node metastasis [24, 27]. Similarly, EGCs with LNM were found to be more in submucosa invasion and LVI in our study. Besides them, tumor differentiation and histologic types have been demonstrated to predict the likelihood of LNM [28, 29]. However, in our study, we failed to found significant differences in the rate of LNM among them, although this trend still existed. The incidence of LNM can approach 31.7% for signet ring cell in contrast with 13.2% for those without LNM. With regard to differentiation, 51.2% were undifferentiated type in LNM group and 24.6% in no LNM group. It may due to the small number of cases with LNM.

The overall survival of patients with EGC is relatively optimistic, and similarly in our study, the overall 1- and 3-year survival rates in LNM group approached 100% and 91.1%, respectively, in contrast with 100% and 100% for those with no LNM. It suggested that LNM was a prognostic factor for 3-year overall survival rate of EGC.

Some limitation of this study should be acknowledged. Firstly, it was a retrospective design and a reviewed data for only surgically resected cases, and not for included patients with EGC who underwent ESD. Secondly, a selection bias may be existed, since all included patients were enrolled at a single academic referral center. Thirdly, we assessed the 1- and 3-year overall survival rates; the rates of 5-year overall and 5-year relapse-free survival also need further evaluation.

## 5. Conclusion

In conclusion, our study demonstrated that EGCs were investigated in 10.0% of gastric cancer, and among EGCs, 12.3% have been proven to be with LNM. We also found five independent predictors of LNM as following: male sex, elderly patients, the depth of invasion, depressed type, tumor located in angle and corpora, and the presence of LVI. The overall 3-year overall survival rate was greater in patients without LNM.

## Figures and Tables

**Table 1:** Clinical characteristics of the included patients.

	Total (*n* = 1004) *n* (%)	LNM (−) (*n* = 881) *n* (%)	LNM (+) (*n* = 123) *n* (%)	*P* value
Mean ages (years ± Sd)	59.65 ± 11.09	60.23 ± 10.88	55.50 ± 11.10	<0.001
≦40	57 (5.7%)	40 (4.5%)	17 (13.8%)	
40–49	139 (13.8%)	116 (13.2%)	23 (18.7%)	
50–59	311 (31.0%)	276 (31.3%)	35 (28.5%)	
≧60	497 (49.6%)	449 (51.0%)	48 (39.0%)	
Male/female	720/284	651/230	69/54	<0.001
*H. pylori* infection	783 (78.0%)	652 (74.9%)	97 (78.9%)	<0.001

**Table 2:** Endoscopic features of the included patients.

	Total (*n* = 1004)	LNM (−) (*n* = 881) *n* (%)	LNM (+) (*n* = 123) *n* (%)	*P* value
Location				<0.001
Cardia	217 (21.6%)	204 (23.2%)	13 (10.6%)	
Angle	124 (12.4%)	104 (11.8%)	20 (16.3%)	
Corpora	207 (20.6%)	176 (20.0%)	34 (27.6%)	
Fundus	10 (0.1%)	8 (0.9%)	2 (1.6%)	
Antrum	446 (44.4%)	389 (44.2%)	54 (43.9%)	
Part of stomach				0.002
Upper part of stomach	240 (23.9%)	224 (25.4%)	16 (13.0%)	
Lower part of stomach	764 (76.1%)	657 (74.6%)	107 (87.0%)	
Lesion size (mm)	19.4 ± 11.7	19.0 ± 11.5	23.0 ± 11.9	<0.001
Small (<2 mm)	506 (50.4%)	464 (52.7%)	42 (34.1%)	
Middle (2-3 mm)	269 (26.8%)	234 (26.6)	35 (28.5%)	
Large (≧3 mm)	229 (22.8%)	183 (20.7%)	46 (37.4%)	
Macroscopic				0.014
Elevated (I + IIa)	93 (9.3%)	80 (9.1%)	13 (10.6%)	
Flat (IIb)	241 (24.0%)	219 (24.9%)	22 (17.9%)	
Depressed (IIc + III)	670 (66.7%)	582 (66.0%)	88 (71.5%)	

**Table 3:** Pathological features of the included patients.

	Total (*n* = 1004)	LNM (−) (*n* = 881) *n* (%)	LNM (+) (*n* = 123) *n* (%)	*P* value
Depth of invasion				<0.001
Mucosa	503 (50.1%)	474 (53.8%)	29 (23.6%)	
Submucosa	501 (50.0%)	407 (46.2%)	94 (76.4%)	
Japanese classification				<0.001
Differentiated	724 (72.1%)	664 (75.4%)	60 (48.9%)	
Undifferentiated	280 (27.9%)	217 (24.6%)	63 (51.2%)	
WTO				<0.001
Well	449 (44.7%)	351 (39.8%)	98 (79.7%)	
Moderately	375 (37.4)	353 (40.1%)	22 (17.9%)	
Poorly	180 (17.9%)	177 (20.1%)	3 (2.4%)	
Histological morphology				<0.001
Adenocarcinoma	793 (79.0%)	719 (81.6%)	74 (60.2%)	
Mucinous carcinomas	48 (4.8%)	39 (4.4%)	9 (7.3%)	
Signet ring cell	155 (15.4%)	116 (13.2%)	39 (31.7%)	
Carcinoma with lymphoid stroma	3 (0.3%)	3 (0.3%)	0 (0%)	
Neuroendocrine carcinoma	5 (0.5%)	4 (0.5%)	1 (0.8%)	
Perineural invasion	7 (0.6%)	6 (0.7%)	1 (0.8%)	0.869
LVI	60 (6.0%)	29 (3.3%)	31 (25.2%)	<0.001
TMN				0.011
Stage 0	914 (91.0%)	881 (100%)	33 (26.8%)	
Stage I	90 (9.0%)	0	90 (73.2%)	

**Table 4:** Multivariate logistic regression analyses for lymph node metastasis of early gastric cancer.

Variables	Multivariate analysis		
	*P* value	OR	95% CI
Male sex	0.035	2.365	1.064–5.257
Age ≦ 40	0.012	0.055	0.006–0.533
Type of depressed	0.013	2.721	1.232–6.008
Submucosa invasion	0.032	2.987	1.100–8.111
LVI	0.003	5.186	1.751–15.366
Location			
Corpora	0.047	8.904	1.029–77.070
Angle	0.024	12.998	1.400–120.677

OR: odds ratio; CI: confidence interval.
